# Gender in cardiac resynchronisation therapy

**DOI:** 10.15171/jcvtr.2018.34

**Published:** 2018-11-10

**Authors:** Tatiana N. Enina, Vadim A. Kuznetsov, Anna M. Soldatova, Tatiana I. Petelina, Dmitriy V. Krinochkin, Alexander Yu. Rychkov, Olga Yu. Nochrina

**Affiliations:** ^1^Scientific researcher in Instrumental Laboratory of Tyumen Cardiology Research Center, Tomsk National Research Medical Center, Russian Academy of Science, Tomsk, Russia; ^2^Director of Tyumen Cardiology Research Center, Tomsk National Research Medical Center, Russian Academy of Science, Tomsk, Russia

**Keywords:** Cardiac Resynchronisation, Therapy, Gender Differences

## Abstract

***Introduction:*** Gender differences in cardiac resynchronisation therapy (CRT) response are not clear enough. This study aimed to assess gender influence on systemic inflammation, neurohormonal activation, fibrosis in patients with congestive heart failure (CHF) and CRT.

***Methods:*** We compared group I (61 men) and group II (16 women) of patients undergoing CRT. Plasma levels of Nt-proBNP, interleukin (IL)-1β, IL-6, IL-10, tumor necrosis factor alpha (TNF-α), C-reactive protein, galectin-3 (Gal-3), metalloproteinase-9 (MMP-9), tissue inhibitors of metalloproteinase 1 and 4 (TIMP-1, TIMP-4), ratio MMP-9/TIMP-1, MMP-9/TIMP-4 were measured. According to dynamics of left ventricular end-systolic volume patients were classified into non-responders, responders, super-responders.

***Results:*** Women more likely had left bundle branch block (81.3 vs 47.5%, *P * = 0.016), were more super-responders (66.7 vs 30.5%). Both groups showed decrease of IL-6 (*P * < 0.05), TNF-α (*P * < 0.001; *P * < 0.05), NT-proBNP (*P * = 0.001; *P * < 0.05), Gal-3 (*P * < 0.05). In women there was decrease of IL-6 by 44.4 vs 23.5% in men (P*P * = 0.029), TNF-α by 41.4 vs 30.9%, NT-proBNP by 73.3 vs 46% (*P * = 0.002), Gal-3 by 82.3 vs 64.9% (*P * < 0.05). Group I also showed decrease of IL-10 by 34.2% (*P * < 0.05). Group dynamics of TIMP-1 was opposite: men showed tendency to reduction of TIMP-1 (*P * = 0.054), women showed increase of TIMP-1 (*P * < 0.05). Besides, men showed decrease of MMP-9 (*P * < 0.05) and ratio MMP-9/TIMP-4 (*P * < 0.05).

***Conclusion:*** The best response to CRT is associated with female gender explained by greater decrease of neurohormonal activation, systemic inflammation and fibrosis. The revealed opposite dynamics of TIMP-1 in the groups can demonstrate the existence of gender features of matrix metalloproteinase system activity and their tissue inhibitors.

## Introduction

Congestive heart failure (CHF) remains highly fatal pathology for men and women.^1^ A modern method for treatment of CHF is cardiac resynchronisation therapy (CRT) which effects lead to remodeling of heart, reduction of symptoms, decrease in hospitalization and mortality.^2-4^ Earlier more efficiency of CRT was shown in women.^5-8^ Among the possible reasons causing gender specifics of remodeling of heart in women, smaller sizes of heart and duration of QRS, higher percent of β-blockers, digoxin, antagonists of an aldosterone,^7^ higher dynamics of remodeling of heart,^6,9,10^ higher percent of biventricular stimulation,^8,10,11^ higher frequency of dilated cardiomyopathy^8,12^ and complete left bundle branch block (CLBBB), higher frequency of atrial fibrillation in men were noted., However, mechanisms of higher CRT efficiency in women are still not clear. It is complicated owing to the low percent of the women included in researches on CRT - about 20%.^8,12-15^ The researches COMPANION (Comparison of Medical Therapy, Pacing, and Defibrillation in Heart Failure) and CARE-HF (Cardiac Resynchronization-Heart Failure) did not confirm gender distinctions of CRT efficiency.^16,17^

An important role in CHF genesis is played by the immune inflammation.^18^ The cytokine system imbalance can result in myocardium remodeling because of matrix metalloproteinase (MMPs) activation causing collagen degradation and remodeling of the extracellular cardiac matrix (ECM),^19^ development of myocardium fibrosis.^20,21^ CRT influence on the processes of inflammation,^22-25^ remodeling of ECM,^26-28^ fiber formation is known.^29,30^ However, influence of these processes on gender specifics of myocardium remodeling after CRT is not studied.

This study aimed to study gender features of CRT efficiency, determine its influence on dynamics of the immune inflammation, neurohormonal activation markers and fibrosis in patients with CHF.

## Materials and Methods

Initially and in the term of the best response to СRT evaluated by the maximum decrease in left ventricular end-systolic volume (LVESV), 77 patients with CHF of ischemic (65%) and non-ischemic genesis from the Register of the performed CRT operations in the Tyumen Cardiology Research Center^31^ were examined. Patients signed the informed consent to comprehensive examination and intervention. Two groups were formed on the gender basis: I group (n = 61; 79%) men, II group (n = 16; 21%) - women. CRT-D devices were implanted in 66.1% of men and in 38.9% of women (р=0.039). The clinical characteristic of groups is presented in Table 1. The groups were comparable by the main parameters, however, women more often had CLBBB (81.3% vs 47.5%, *P* = 0.016), and men – atrial fibrillation (39% vs 17%, *P* = 0.017). No differences in therapy in the groups were noted.

**Table 1:** Clinical characteristic of patients

**Parameter**	**Group I** **men (n=61)**	**Group II** **women (n=16)**	***P***
Time of the best response to CRT, month	14.0 [4.5;32.0]	16.0 [10.0;30.0]	NS
Age (y)	55.7±7.8	56.3±10.2	NS
CAD (%)	42 (68.9)	8 (50)	NS
PMI (%)	25 (41.0)	3 (18.8)	NS
CABG (%)	8 (13.1)	0	0.099
PCI (%)	16 (26.2)	2 (12.5)	NS
FC II (%)	24 (39.3)	7 (43.8)	NS
FC III (%)	31 (50.8)	6 (37.5)	NS
FC IV (%)	6 (9.8)	3 (18.8)	NS
AH (%)	46 (75.4)	13 (81.2)	NS
DM (%)	9 (14.8)	3 (18.8)	NS
Obesity (%)	41 (67.2)	13 (81.3)	NS
AF (%)	23 (39)	3 (17)	0.017
RFA AV-connections (%)	25 (41.0)	3 (18.8)	NS
CLBBB (%)	29 (47.5%)	13 (81.3%)	0.016
Nitrates (%)	30.5	22.2	NS
Ca channel blockers (%)	20.3	27.8	NS
Digoxin (%)	61.0	38.9	0.098
Statins (%)	79.7	72.2	NS
β-blockers (%)	100.0	100.0	NS
Diuretics (%)	98.3	100.0	NS
MCRA (%)	100.0	100.0	NS
Warfarin (%)	50.8	27.8	NS
ACEI (%)	93.2	77.8	0.060
Antiarrhythmics (%)	47.5	50.0	NS
Response to CRT			0.003
Non-responders (%)	22 (36.1)	3 (18.8)	
Responders (%)	19 (31.1)	3 (18.8)	
Super-responders (%)	20 (32.8)	10 (62.4)	

CRT, cardiac resynchronisation therapy; CAD, coronary artery disease; PMI, previous myocardial infarction; CABG, coronary artery bypass grafting; PCI, percutaneous coronary intervention; FC, functional class due to NYHA classification; AH, arterial hypertension; DM, diabetes mellitus; AF, atrial fibrillation; RFA AV-connection, radiofrequency ablation of atrioventricular connection; CLBBB, complete left bundle branch block; MCRA, mineralocorticoid receptor antagonists; ACEI, angiotensin-converting enzyme inhibitors; NS, not significant (*P* > 0.05).

The functional class of heart failure was defined taking into account 6-minute walking test. Echocardiography (EchoCG) was performed in dynamics using IE 33 (Philips). Plasma NT-proBNP levels, interleukin (IL) - 1b, IL-6, IL-10, FNO-α, galectin-3 (Gal-3), matrix metalloproteinase 9 (MMR-9), tissue inhibitors of metalloproteinase (TIMP-1 and TIMP-4) were analyzed by the method of solid-phase chemiluminescent immunoassay (a “sandwich-method”) on the IMMULITE 1000 analyzer (Siemens Diagnostics, the USA). Coefficients of MMP-9/TIMP-1, MMP-9/TIMP-4 were calculated. Determination of highly sensitive C-reactive protein in blood serum was carried out by the immune turbidimetric method using analytical PROTEIN C- REACTIVE sets (BioSystems, Spain) on the Clima MC-15 analyzer (Spain).

In normal distribution of the data, the results are presented as a mean value* ±* standard deviation, in abnormal distribution – as a median and interquartile range [25%, 75%]. Quantitative data were compared by t-Student criterion, in abnormal distribution - in case of intergroup comparison Mann-Whitney U-criterion was used, in case of intra group - Wilcoxon criterion. Qualitative variables compared by criterion χ². *P* < 0.05 was considered to be significant.

## Results

In both groups, there was a positive dynamics of echocardiographic parameters after CRT (Table 2). However, the rate of change of some parameters was more significant in the group of women (Table 3). In group II more super-responders (62.5 vs 32.8%) were registered, less nonresponders (18.8 vs 36.1%) and responders (18.8 vs 31.1%) (*P* = 0.03) (Table 1). Significant decrease of IL-6 (*P* < 0.05), TNF-α (*P* < 0.001 in group I; *P* < 0.05 in group II) (Table 4), NT-proBNP (*P* = 0.001; *P* < 0.05), Gal-3 (*P* < 0.05) was observed in both groups (Table 5). However, in group II dynamics of biomarkers was more expressed: IL-6 decrease by 44.4 vs 23.5% in group I (*P* = 0.029), TNF-α by 41.4 vs 30.9%, NT-proBNP by 73.3 vs 46% (*P* = 0.002), Gal-3 by 82.3 vs 64.9%. In group I significant degrease of IL-10 by 34.2% (*P* < 0.05), MMP-9 (*P* < 0.05) and MMP-9/TIMP-4 was registered (*P* < 0.05). Opposite dynamics of TIMP-1 was found in both groups: in group I – tendency to TIMP-1 decrease (*P* = 0.054), in group II – significant TIMP-1 increase (*P* < 0.05) (Table 5).

**Table 2:** EсhoCG parameters in groups

**Parameter**		**Group I** **men (n=61)**	**Group II** **women (n=16)**	**P between groups**
АО (mm)		35.2±3.0	31.7±3.0	<0.001
LA (mm)	Initially	51.2±6.5	47.7±6.3	0.049
	In dynamics	48.8±5.8*	43.5±7.6*	0.015
RA (ml)	Initially	89.2±32.4	60.6±33.2	0.006
	In dynamics	76.8±31.4*	54.6±30.1#	0.084
RV (mm)	Initially	31.2±4.0	28.2±4.4	0.013
	In dynamics	29.6±3.9*	26.2±2.7#	<0.001
LVESD (mm)	Initially	55.4±7.0	52.1±5.8	0.064
	in dynamics	51.1±9.3*	44.4±4.9*	0.006
LVEDD (mm)	Initially	65.8±7.2	62.6±6.0	0.062
	In dynamics	62.5±7.6*	55.8±3.8*	<0.001
LVESV (ml)	Initially	154,5±46.5	135.8±35.0	0.075
	In dynamics	117.6±49.4*	78.7±26.1*	<0.002
LVEDV (ml)	Initially	225.4±56.1	200.4±42.4	0.050
	In dynamics	190.5±59.2*	142.8±37.3*	<0.001
LVEF (%)	Initially	32.1±6.3	32.8±5.3	NS
	In dynamics	39.6±8.8*	45.5±7.3*	0.008
Dyssynergia LV (%)		19.0±24.0	2.2±6.7	0.098

* *P* < 0.001 in the group; ^#^*P* < 0.05 in the group; NS, not significant; LA, left atrium; RA - right atrium; RV, right ventricle; LV, left ventricle; LVESD, left ventricular end systolic diameter; LVEDD, left ventricular end diastolic diameter; LVESV, left ventricular end systolic volume; LVEDV, left ventricular end diastolic volume; LVEF, left ventricular ejection fraction.

**Table 3:** Degree of EсhoCG changes in the groups

**Parameter**	**Group I** **men (n=61)**	**Group II** **women (n=16)**	***P*** **between groups**
∆LA (mm)	-2.4±3.9	-4.2±3.1	0.057
∆RA (mm)	-14.9±21.5	-22.8±19.9	NS
∆RV (ml)	-1.6±2.9	-2.0±3.0	NS
∆LVESD (mm)	-4.8±4.7	-7.3±6.4	0.097
∆LVEDD (mm)	-3.3±4.1	-6.8±4.3	0.005
∆LVEDV (mL)	-35.0±35.2	-57.6±40.7	0.043
∆LVESV (mm)	-37.0±28.8	-57.0±33.1	0.035
∆LVEF (%)	7.5±8.2	12.7±8.6	0.031

**Table 4:** Inflammation markers in the groups

**Parameter**	**Group I** **men (n=61)**	**Group II** **women (n=16)**	***P*** **between groups**
IL-1β (pg/ml)	4.1[3.4;4.8]	4.1[3.8;4.3]	NS
IL-1β in dynamics (pg/mL)	3.4[3.1;4.3]	4.4[3.2;5.0]	NS
IL-6 (pg/mLl	3.4[2.6;6.6]	2.7[2.5;3.9]	NS
IL-6 in dynamics (pg/mL)	2.6[1.6;4.6]#	1,5[1.2;3.0]#	0.029
IL-10 (pg/mlL)	4.1[2.7;5.0]	3.0[1.7;5.0]	NS
IL-10 in dynamics (pg/mL)	2.7[2.1;4.1]#	2.5[1.8;3.0]	NS
TNF-α (pg/mL)	9.4[8.0;11.2]	9.9[6.9;11.0]	NS
TNF-α in dynamics (pg/mL)	6.5[4.9;9.0]*	5.8[4.5;8.6]#	NS
CRP (mg/mL)	4.8[2.4;9.1]	4.1[2.0;6.6]	NS
CRP in dynamics (mg/mL)	3.1[1.6;6.7]	2.1[0.8;6.6]	NS

# *P* < 0.05; * - *P* < 0.001; IL - interleukin; TNF-α - tumor necrosis factor alpha; CRP - C-reactive protein

**Table 5:** NT-proBNP and markers of fibrosis in the groups

**Parameter**	**Group I** **men (n=61)**	**Group II** **women (n=16)**	***P*** **Between groups**
NT-proBNP (pg/ml)	2134.5 [1010.3;4156.8]	1154.0 [537.0;2485.5]	0.003
NT-proBNP in dynamics (pg/ml)	1153.0 [582.0;2285.5]**	308.5 [237.0;568.3]*	0.002
Galectin-3 (ng/ml)	0.74 [0.28;12.3]	0.62 [0.14;1.1]	NS
Galectin-3 in dynamics (ng/ml)	0.26 [0.04;1.36]*	0.11 [0.04;0.31]*	NS
ММР-9 (ng/ml)	157.9 [121.3;203.5]	129.1 [83.5;303.5]	NS
ММР-9 in dynamics (ng/ml)	136.5 [113.6;157.4]*	132.4 [88.4;153.0]	NS
ТIМР-1 (ng/ml)	471.5 [330.1;570.0]	360.9 [271.9;405.4]	0.038
TIMP-1 in dynamics (ng/ml)	413.8 [333.9;485.2]§	410.6 [275.0;461.0]*	NS
TIMP-4 (ng/ml)	2131.5 [1634.0;3252.2]	2185.1 [1865.3;2611.7]	NS
TIMP-4 in dynamics (ng/ml)	2516.4 [1808.5;3416.6]	2549.3 [2116.9;3260.5]	NS
MMP-9/TIMP-1 (U)	0.34 [0.25;0.51]	0.38 [0.23;0.63]	NS
MMP-9/TIMP-1 in dynamics (U)	0.35 [0.25;0.49]	0.35 [0.26;0.47]	NS
MMP-9/TIMP-4 (U)	0.07 [0.05;0.12]	0.06 [0.03;0.11]	NS
MMP-9/TIMP-4 in dynamics (U)	0.05 [0.03;0.07]*	0.05 [0.03;0.08]	NS

Abbreviations: NT-proBNP, N-terminal fragment of pro-brain natriuretic peptide; MMP, matrix metalloproteinase; TIMP, tissue inhibitor of matrix metalloproteinase. * *P* < 0.05; ** *P* = 0.001; § *P* = 0.054.

## Discussion

Association of positive reaction to CRT with the female gender can be due to significant decrease in neuro-humoral activity and immune inflammation. NT-proBNP significant decrease in patients with the favorable reaction to CRT was already described.^32-35^ Both initially and in dynamics lower concentrations of NT-proBNP were found in group II, which is probably connected with the smaller size of cardiac cavities in women, and also with larger dynamics of the return remodeling after CRT (Tables 2 and 3).

Association of CRT efficiency with the activity of immune inflammation was described by a number of authors.^23-25^ Unlike our results, Boriani et al. in 3 months,^36^ Osmancik et al. in 6 months^24^ Seifert et al^37^ and Tarquini et al in 1 year of CRT^34^ did not reveal IL-6 and TNF-α dynamics. At the same time Orrego et al in 3 months of CRT noted significant TNF-α decrease.^38^ Rordorf et al^23^ showed TNF-α influence on remodeling of cardiac cavities after CRT. TNF-α is one of the main mediators of the immune response. Cardiomyocytes can induce TNF-α in myocardium wall tension (a diastolic stress), and the higher final diastolic pressure level in left ventricle, the higher amount of the produced cytokine.^39^ Significant decrease of cardiac cavities in group II can explain the possible mechanism of the more expressed TNF-α decrease. TNF-α, IL-6, IL-1β common property is their ability to exert negative inotropic effect. Naturally, the more expressed decrease of cytokine concentration in group is followed by higher dynamics of EchoCG and increase of left ventricular ejection fraction. The fixed terms of CRT efficiency assessment without considering its best efficiency according to LVESV dynamics, used in our work, can influence the discrepancy of results in researches. In the absence of differences IL-6 basic levels in groups, its dynamics was more expressed in group II. Development of LV diastolic dysfunction ^40^ is associated with IL-6. In researches decrease of IL-6 decrease, TNF-α was described only in responder.^24,32^ The higher percent of patients with the favorable reaction to CRT in group II is naturally followed by higher cytokine level decrease.

Immune inflammation is known to influence the processes of a fiber formation and remodeling of ECM. Gal-3 is a rather new marker of fibrosis, which increased concentration are followed by progressing diastolic dysfunction. The study of MADIT-CRT showed that the increased Gal-3 level is an independent predictor of an unfavourable outcome in patients with moderate CHF.^29^ However, CARE-HF studies showed no Gal-3 level changes after CRT.^30^ In BIOCRT study predictor ability of GAL-3 in the complex with NT-proBNP and sST2^41^ was compared. The increased levels of all three studied biomarkers in the coronary sinus showed 95% specificity for the negative response to СRT. Our results confirm Gal-3 level decrease after CRT in the groups.

There are data on CRT influence on MMPs system activity and their tissue inhibitors (TIMPs) playing a key role in ECM remodeling.^26-28^ ECM modernization is in fact an adaptive process leading to changes of myocardium tensile properties, preservation of heart geometrical shape or formation of cardiac chambers new formation. MMPs and TIMPs both localized in the myocardium regulate synthesis and disintegration of ECM proteins, interfering with the development of disorders of systolic and diastolic functions. There are literature data on cytokine direct influence on the ECM catabolism activity by MMPs activation.^42^ The results of our study confirm CRT ability to exert modulating influence on ECM due to the decrease in MMP-9 and the ratio of MMP-9/TIMP-4 in the group of men. However, the revealed opposite dynamics of TIMP-1 levels in the groups may show the existence of gender specifics of ECM remodeling. Tolosana et al in 12 months of CRT in the group of non-responder noted higher levels of fibrosis markers (MMP-2 and TIMP-1), and TIMP-1 level ≥248 ng/mL was an independent predictor of non-responders to CRT with 71% of sensitivity and 72% of specificity.^29^ In the groups studied higher basic TIMP-1 levels were found, moreover in group II its significant increase in dynamics was registered. In spite of this 62.4% of women became super-responders, and 18.8% - responder, thus allowing to consider fiber formation process as an adaptive, promoting preservation of heart chambers geometry. Perhaps, TIMP-1 level increase in group II may confirm stabilization of ECM remodeling, in which collagen synthesis is higher than its disintegration in immune inflammation activity decrease.

Distinctions of response to CRT may be due to biological differences between men and women. Studying of sex hormones was not the task of the present research. However, cardioactive effects of sex hormones on physiological processes in myocardium may influence CRT outcomes. Literature describes ability of sex hormones to exert both anti-inflammatory effect,^43^ and cardioprotective action on ECM state.^44-46^ Further studies are necessary.

Thus, the best response to CRT is associated with women that is probably caused by a more marked decrease of system inflammation, neurohormonal activation, fibrosis. CRT probably exerts the modulating effect on ECM state by decrease of system inflammation which plays the leading role in heart remodeling. The revealed opposite dynamics of TIMP-1 in the groups can demonstrate the existence of gender features of matrix metalloproteinase system activity and their tissue inhibitors.

## Ethical approval

This investigation has been approved by the Ethics Committee of the Tyumen Cardiology Research Center.

## Competing interests

All authors declare no competing financial interests exist.
